# Deactivation of Cellulase at the Air-Liquid Interface Is the Main Cause of Incomplete Cellulose Conversion at Low Enzyme Loadings

**DOI:** 10.1038/s41598-018-19848-3

**Published:** 2018-01-22

**Authors:** Samarthya Bhagia, Rachna Dhir, Rajeev Kumar, Charles E. Wyman

**Affiliations:** 1 Department of Chemical and Environmental Engineering, Bourns College of Engineering, University of California Riverside, 900 University Ave, Riverside, CA 92521 USA; 2 Center for Environmental Research and Technology, Bourns College of Engineering, University of California Riverside, 1084 Columbia Ave, Riverside, CA 92507 USA; 3 BioEnergy Science Center (BESC), Oak Ridge National Laboratory, PO Box 2008 MS6341, Oak Ridge, TN 37831 USA

## Abstract

Amphiphilic additives such as bovine serum albumin (BSA) and Tween have been used to improve cellulose hydrolysis by cellulases. However, there has been a lack of clarity to explain their mechanism of action in enzymatic hydrolysis of pure or low-lignin cellulosic substrates. In this work, a commercial *Trichoderma reesei* enzyme preparation and the amphiphilic additives BSA and Tween 20 were applied for hydrolysis of pure Avicel cellulose. The results showed that these additives only had large effects on cellulose conversion at low enzyme to substrate ratios when the reaction flasks were shaken. Furthermore, changes in the air-liquid interfacial area profoundly affected cellulose conversion, but surfactants reduced or prevented cellulase deactivation at the air-liquid interface. Not shaking the flasks or adding low amounts of surfactant resulted in near theoretical cellulose conversion at low enzyme loadings given enough reaction time. At low enzyme loadings, hydrolysis of cellulose in lignocellulosic biomass with low lignin content suffered from enhanced enzyme deactivation at the air-liquid interface.

## Introduction

Cellulases, such as those secreted extracellularly by the industrial strain of *Trichoderma reesei*, a soft-rot fungus, find applications in cellulosic ethanol, pulp and paper, textiles, food, and agriculture industries due to their ability to cleave glycosidic bonds in cellulose at low temperatures at environmentally safe conditions^1^. However, these enzymes are relatively expensive, lose their activity over time of reaction, and cannot be recycled efficiently from the solid-liquid reaction medium^2^. A typical example is that with 5 mg protein per gram cellulose loading of commercial preparation of DuPont Accellerase^®^ 1500 at 50 °C and at pH 5.0 after 5 days of reaction, only 50% conversion of pure Avicel cellulose (PH-101 grade) to glucose could be realized^3^. Cellulose conversions at the low cellulase loadings required for commercial application, however, are even lower for real lignocellulosic biomass^4^. Therefore, there is an acute need to improve cellulose conversions at low enzyme loadings to improve the return on products derived through reactions catalyzed by cellulase. Non-ionic surfactants such as polyethoxylated sorbitan ester type surfactants, commonly known as Tween^®^^5^ and non-catalytic proteins like bovine serum albumin (BSA)^6,7^ are known to improve cellulose conversions from enzymatic hydrolysis of pretreated lignocellulosic biomass. Two key studies, one by Eriksson *et al*.^8^ on surfactants and BSA in 2002 and the other by Yang and Wyman^9^ on BSA in 2006, showed that the mechanism by which surfactants or BSA improve cellulose conversions is through reducing or preventing adsorption of enzyme to lignin. This important discovery came from the learning that while large increases in cellulose conversions and rates were seen in enzymatic hydrolysis of lignocellulosic biomass with surfactant or BSA addition, these additives did not increase cellulose conversions and rates in substrates devoid of lignin (Avicel, or delignified lignocellulosic biomass). It was also seen that surfactants or BSA had no effect on adsorption of cellulase to Avicel while they reduced adsorption of cellulase onto lignin in lignocellulosic biomass. Now, while these studies found no effect of surfactants or BSA on lignin-free cellulosic substrates such as Avicel, several articles from the past century show large effect of surfactants on hydrolysis of pure cellulosic substrates such as Avicel, cotton, and newspaper by cellulases recovered from *T. reesei* and other wood-rot fungi^10–14^. Despite extensive research, it has not been clear why some studies showed no effect, while others showed significant effect of surfactants on enzymatic hydrolysis of pure cellulosic substrates. Thus, the purpose of this study was to better understand the mechanism of surfactants and BSA in enhancing hydrolysis of pure cellulosic substrates by cellulases. Improving our knowledge of their impact on enzymatic hydrolysis is of extreme importance to help define paths to reduce cellulase costs for industries that employ cellulases, especially ones targeting production of renewable fuels and chemicals from renewable lignocellulosic biomass.

Several mechanisms have been put forward to explain the positive effect of surfactants on hydrolysis of lignin-free (pure) cellulosic substrates by enzymes from wood-rotting fungi. Earliest records date to 1952 when D. R. Whitaker^15^ proposed that this was due to stimulation of cellulase adsorbed onto insoluble cellulose substrates by BSA. Elwyn T. Reese^10^ in 1980 proposed that shear stress caused deactivation of cellulase due to shaking as enzymes were stable in absence of shaking. He proposed that in presence of cellulose, cellulases expose their hydrophobic groups and denature due to aggregation through hydrophobic binding at shaken conditions. He further noted that formation of protein-surfactant complexes reduces this aggregation, thus protecting the enzyme. Castanon and Wilke^11^ in 1981 proposed that surfactants alleviate immobilization of enzymes on the surface of insoluble cellulosic substrates to prevent them getting jammed or stuck. In a symposium in 1980^16^, Reese and co-workers hypothesized that certain compounds may protect enzymes from shear or air-liquid interface inactivation under shaking conditions. In 1982, they^17^ found that deactivation of enzymes was severe at the air-liquid interface along with shear stress rather than shear stress alone. Ooshima *et al*. in 1986^18^ proposed that surfactants perturb the exoglucanase-endoglucanase adsorption ratio on the solid substrate surface to favor enzymatic hydrolysis and simultaneous saccharification and fermentation (SSF). Helle *et al*.^13^ in 1993 suggested that surfactants possibly modify the surface of cellulose substrates and reduce inactivation of adsorbed enzyme (enzyme immobilization). Apart from these mechanisms, it is also known that BSA^19^ and non-ionic surfactants^20^ are used to reduce nonspecific binding of proteins or peptides on solid vessel surface. It is also possible that BSA or surfactants shield enzymes from product inhibition by glucose or protect against heat deactivation due to long time reactions at 50 °C. Thus, a total of ten possible mechanisms can be proposed to explain how surfactants could increase cellulose conversion: stimulation, lower shear stress, protection through enzyme-surfactant complex, lower enzyme immobilization on cellulose, reduced loss at the air-liquid interface, altered exoglucanase-endoglucanase adsorption ratio, substrate modification, reduced loss of enzyme on reaction vessel walls, lower glucose inhibition, and reduced heat deactivation.

Our investigation involved Avicel as a model cellulosic substrate that contained 97% cellulose and 3% xylan by weight as determined by compositional analysis of biomass^21,22^. The DuPont’s Accellerase^®^ 1500 cellulase enzyme preparation employed had a protein content of 82 mg/ml as determined by bicinchoninic acid (BCA) assay^23^. The filter paper activity (FPU) of this preparation was 0.5 FPU/mg, reported elsewhere^24,25^. First, the enzyme to substrate ratio was varied in the presence of surfactant to define their effect on cellulose conversion. Then, the effects of BSA, Tween 20, and defatted soybean flour were compared to determine if proteins and surfactants acted differently. The possibility of amphiphilic additives working through prevention of nonspecific binding on glass surface was investigated by comparing cellulose conversions in borosilicate glass, polycarbonate, and siliconized Erlenmeyer glassware. Experiments without shaking were performed and substrate addition delayed to determine the relevance of these effects in altering conversion. Then the air-liquid interfacial area of the reaction vessel was varied while keeping the volume constant to understand the effect of air on cellulose conversion. Lastly, solids with low and high lignin content produced by poplar pretreatment were hydrolyzed at low enzyme loading with surfactant to determine the impact of surface deactivation on enzymatic conversion of lignocellulosic biomass into fermentable sugars. Nearly 130 combinations that included other cellulosic substrates, biomass substrates, another commercial enzyme preparation (Novozymes Cellic^®^ CTec2), different substrate and surfactant loadings, and several environmental conditions were carried out. This first report on this subject reveals that surface tension at the air-liquid interface was primarily responsible for cellulase deactivation resulting in low conversions at low cellulase loadings. Additional findings on this work beyond what can be covered here will be published later.

## Results and Discussion

**Figure 1:**
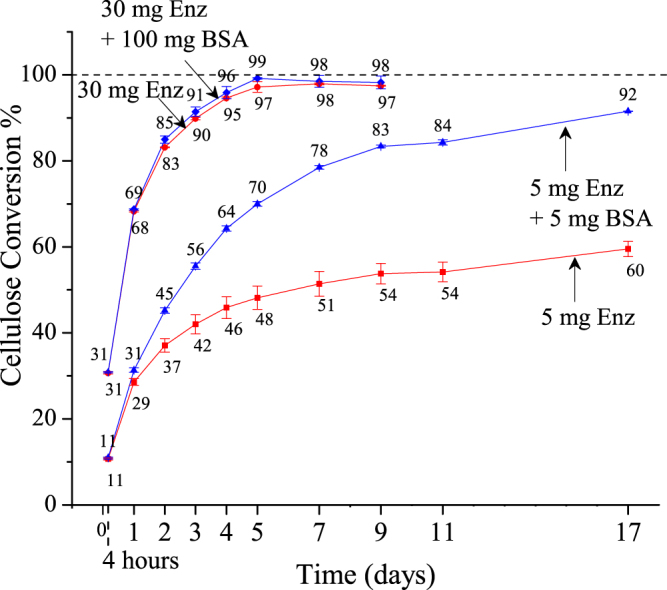
Effect of BSA at low and high cellulase loadings on Avicel cellulose conversion. Low enzyme loading curves show cellulose conversion for up to 17 days of enzymatic hydrolysis of Avicel (1% glucan loading) with 5 mg enzyme (Accellerase^®^ 1500) (square) and 5 mg enzyme with co-addition of 5 mg BSA (triangle). High enzyme loading curves show cellulose conversion for up to 9 days of enzymatic hydrolysis of Avicel (1% glucan loading) with 30 mg enzyme (Accellerase^®^ 1500) (circle) and 30 mg enzyme with co-addition of 100 mg BSA (diamond). Enzyme and BSA loadings were based on mg per gram glucan in substrate. Error bars represent standard deviation from three replicate flasks.

**Figure 2:**
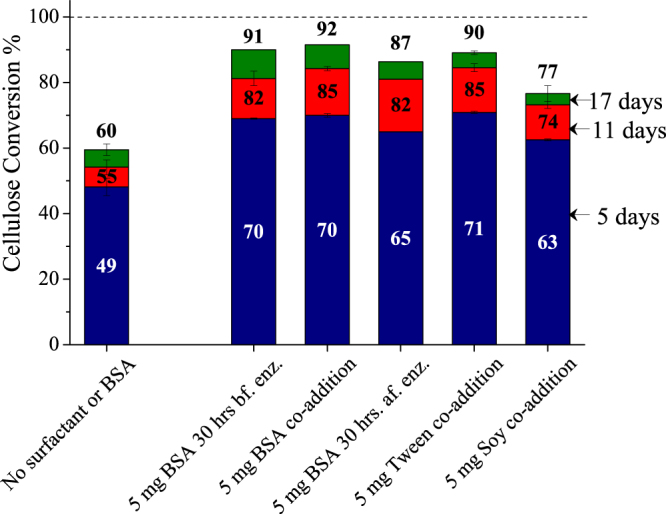
Comparison of BSA, Tween, and soy flour on enzymatic hydrolysis of Avicel. From left to right, columns show cellulose conversion after 5, 11, and 17 days of enzymatic hydrolysis of Avicel (1% glucan loading) with 5 mg enzyme (Accellerase^®^ 1500), with supplementation of 5 mg BSA 30 hours before enzyme addition, co-addition of 5 mg BSA, with supplementation of 5 mg of BSA 30 hours after enzyme addition, with co-addition of 5 mg Tween 20, and with co-addition of 5 mg soy flour, added based on per gram glucan in the substrate. Error bars represent standard deviation from three replicate flasks.

Conditions similar to those applied by Yang and Wyman^9^ of high enzyme and BSA loading were applied first to Avicel cellulose to confirm the effect of BSA on conversion. Figure 1 shows that 30 mg of enzyme achieved nearly complete cellulose conversion to glucose and minor amounts of cellobiose, with both represented in their anhydrous forms. Throughout this paper, all loadings of enzyme preparations or additives (BSA or Tween 20) are expressed in milligrams per gram glucan in the substrate. Furthermore, the term enzyme refers to the commercial Accellerase^®^ 1500 cellulase preparation. Addition of 100 mg BSA added simultaneously to 30 mg enzyme had no effect on rates or final cellulose conversion. However, addition of 5 mg of BSA had a large positive effect on conversion of Avicel at low enzyme loading of 5 mg. For this low cellulase loading, although BSA had no effect on initial reaction rates, beneficial effects started to appear after 24 hours of reaction, with an ultimate 40 percent absolute increase in cellulose conversion after 17 days. The fact that its impact was only apparent at low enzyme loadings and not seen until after 24 hours of reaction explains why it was missed in prior studies with high enzyme loadings or experimentation at shorter durations. Because Fig. 2 shows that BSA and Tween 20, a non-ionic surfactant, had the same effect, the amphiphilic nature of BSA appears important to enhancing performance. Since BSA and Tween 20 have a different hydrophilic-hydrophobic balance (HLB), their equivalent effect on cellulose conversion suggests that a stimulation mechanism is unlikely. Moreover, stimulation would possibly need an allosteric enzyme site that is inconsistent with the very different structures of BSA and Tween 20.

Soy flour was also employed to take advantage of the lower cost of high protein soybean meal of $324 to $490 per metric ton over the last three years, as reported by the October 2016 USDA Feed Outlook^26^. It is also compatible with microbes in subsequent ethanol fermentations^27^. Soy flour addition also increased cellulose conversion at the low enzyme loading. Soy flour contains soy lecithin (mixture of phospholipids), soy protein, and soy saponin that exhibit surfactant behavior^28^. However, although the exact soluble surfactant concentration in the complex mixture was unknown, the low solubility of a large portion of soy flour in water likely resulted in the smaller increase in cellulose conversion by enzymes than seen with addition of BSA or Tween 20. Overall, we expect any additive with amphiphilic properties that does not denature enzyme should be able to cause this effect, as evidenced by similar observations of enhanced enzymatic hydrolysis of cellulose by polyethylene glycols^29^, β-lactoglobulin, pepsin, lysozyme, gelatin, peptone^15^, Tweens^18^, Pluronics, Zonyl, polypropylene glycol, polyvinyl pyrrolidone, Triton, Digitonin, methocyl, quarternary ammonium compounds^10^, sophorolipid, rhamnolipid, bacitracin^13^, lecithins, phospholipids, and even cattle saliva^30^ that contains muocproteins and saponins^31^. This diverse range includes cationic, anionic, zwitterionic, and non-ionic surfactants, as well as compounds that cannot be strictly classified as surfactants as they do not form association colloids, and are hence termed as amphiphilic additives^32^. This study focused on finding the underlying cause of cellulase deactivation using the most popular additives Tween and BSA rather than comparing different classes of these additives. Due to their strong electrostatic interactions with proteins, charged surfactants like sodium dodecyl sulfate (SDS) can denature proteins at low concentrations. On the other hand, nonionic and zwitterionic surfactants with overall neutral charge, in most cases, do not denature proteins^33^. Ooshima *et al*.^18^ reported that cationic Q-86W and anionic Neopelex F-25 surfactants denatured cellulase above 0.008% and 0.001%, respectively, while zwitterionic Anhitol 20BS and nonionic Tween 20 did not.

**Figure 3:**
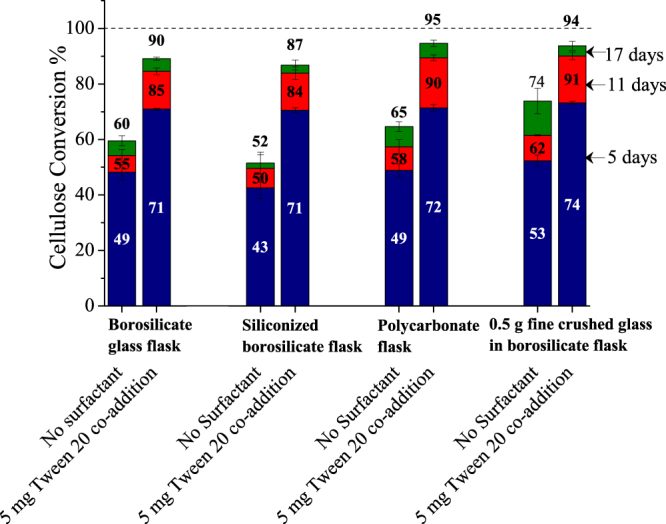
Effect of reaction vessel surface and shear on enzymatic hydrolysis of Avicel. Columns show cellulose conversion after 5, 11, and 17 days of enzymatic hydrolysis of Avicel (1% glucan loading) with 5 mg enzyme (Accellerase^®^ 1500) and with co-addition of 5 mg Tween 20 added based on per gram glucan in substrate, in borosilicate glass flasks, siliconized glass flasks, polycarbonate flasks, and borosilicate glass flasks containing 0.5 grams of glass powder. Error bars represent standard deviation from three replicate flasks.

Figure 2 shows that there were little differences in cellulose conversion through addition of BSA 30 hours before addition of enzyme, co-addition of enzyme and BSA, or BSA addition 30 hours after enzyme. Since BSA and Tween 20 show similar effects, further experiments were carried out with Tween 20 as it is less expensive for commercial use. It is also compatible with ethanologenic organisms like *Saccharomyces cerevisiae*^34^ and *Zymomonas mobilis*^35^. Figure 3 shows that changing the reaction vessel from glass (hydrophilic, water contact angle: 16°)^36^ to polycarbonate (fairly hydrophobic, water contact angle: 70°)^37^ increased cellulose conversion by 5 percentage points at the end of reaction, whereas siliconized glass (hydrophobic, water contact angle: 90°) reduced it by 8 percentage points^36^. This small variation in cellulose conversions with change of reaction vessel material indicated that avoiding enzyme denaturation at the solid surface could not account for the benefits seen with the additives. Interestingly, Tween 20 addition did not reduce these variations. These data point to an enzyme binding mechanism not solely dependent on the hydrophobic or hydrophilic character of the vessel material but that could be influenced by differences in electrostatic charge^38^ or microscopic surface roughness^39^. In any case, since the change in cellulose conversion due to nonspecific binding of enzyme on the solid vessel surface was relatively small, it cannot account for large increase caused by amphiphilic additives as shown in Fig. 3.

To evaluate the role of shear stress might, 0.5 g of fine glass powder, made by crushing a 20 ml glass serum vial with a hammer in a plastic bag, was added to the glass flasks before hydrolysis. Apart from increasing the shear stress, the powered glass greatly increased the contact area between enzyme and glass. Although it was expected that the greater shear stress resulting from shaking the glass powder would severely reduce cellulose conversion, conversion surprisingly increased from 60 to 74%. Furthermore, in the presence of Tween 20 with crushed glass, conversion increased even more to 94%. This outcome ruled out that Tween 20 reduced enzyme deactivation due to shear stress but showed these enzymes were robust even in the presence of sharp surfaces. Thus, it was hypothesized that the increase in cellulose conversions in the presence of crushed glass resulted from particle size reduction and consequently increased Avicel surface area. Reese and co-workers^40^, on the other hand, observed a large effect of shear stress, as their experimentation involved a flowthrough-type apparatus in which flow rate was changed to affect shear stress in glass capillary tubing so that shear rate could be estimated by the Hagen-Poiseuille equation^41^. However, Erlenmeyer shaken flasks do not produce such high shear rates. Furthermore, the glass powder experiments above reveal these cellulases are robust even in the presence of moving sharp glass surfaces.

**Figure 4:**
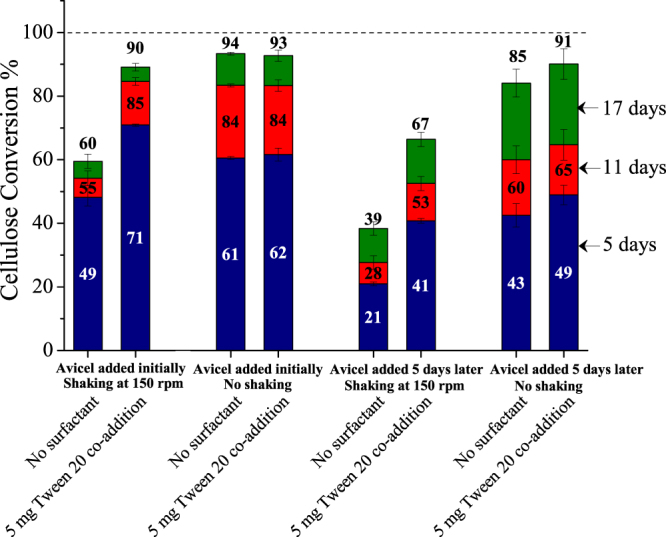
Effect of surfactant on enzymatic hydrolysis of Avicel with and without shaking. Columns show cellulose conversion after 5, 11, and 17 days of enzymatic hydrolysis of Avicel (1% glucan loading) with 5 mg enzyme (Accellerase^®^ 1500) and with co-addition of 5 mg of Tween 20 added based on per gram glucan in the substrate, with and without shaking at 150 rpm. Avicel was either present initially or added 5 days after incubation of enzyme or enzyme with surfactant in buffer solution. For Avicel added 5 days later, days were counted after Avicel was added to the flask. Error bars represent standard deviation from three replicate flasks.

Since surfactants are surface-active compounds, experiments were performed without shaking. Figure 4 shows that cellulose conversion with surfactant and shaking was similar to cellulose conversion without shaking and without surfactant after 17 days, although the reaction rate was slower for the latter case. There was no effect of surfactants on cellulose conversion without shaking. This result strongly indicated an air-liquid interface mechanism. The National Renewable Energy Laboratory (NREL) standard procedure “Enzymatic Saccharification of Lignocellulosic Biomass”^42^ mentions that the shaking rate (rpm) of the reaction flasks should be chosen to maintain solids suspension. Because we observed that Avicel at 1% loading in 50 ml reaction volume was suspended at around 125 rpm in a 125 ml Erlenmeyer flask, all of our shaking experiments were carried out at 150 rpm. However, the fact that cellulose conversions were higher without shaking than with shaking at 150 rpm at low enzyme loading strongly suggested that maintaining a suspension is not always beneficial for achieving high cellulose conversions. Flasks are shaken to improve mass-transfer and reduce localized sugar concentrations that otherwise can be inhibitory to enzymes. However, while shaking increases reaction rates, it also deactivates enzyme. Furthermore, activity loss by shaking should become more significant at low enzyme loadings, resulting in lower cellulose conversion at the longer reaction times needed at low loadings. On the other hand, this observation is not consistent with earlier proposed mechanisms that attribute activity loss to formation of a protein-surfactant complex^10^, protection against thermal deactivation, or less product inhibition as these possibilities should not be influenced by whether the reaction medium was shaken or not. Likewise, as hypothesized in substrate dependent mechanisms, enzyme immobilization due to jamming of cellulase^11^, cellulose modification by change in pore size^13^, or change in exoglucanase-endoglucanase adsorption ratio on cellulose^18^ should affect cellulose conversions in the presence of surfactants even if the flasks were not shaken. Based on the results that deactivation was much more severe when air-bubbles were entrapped in a flowthrough capillary column than by shear stress alone, Reese and co-workers^17^ hypothesized that the air-liquid interface plays a critical role in cellulases deactivation.

Our results with conventional hydrolysis in Erlenmeyer flasks are consistent with the hypothesis that the air-liquid interface alone was decisive in cellulase deactivation. However, to further rule out substrate dependent mechanisms, Avicel cellulose was added to the reaction flasks five days after shaking was started with just enzymes present. Figure 4 shows that cellulose conversions with or without surfactants were lower for shaking flasks to which substrate was added after 5 days. This result is consistent with a significant fraction of enzymes losing activity due to environmental factors when there was no substrate present initially. On the other hand, if Avicel and enzymes are added together as in conventional shaking experiments, cellulase adsorption onto cellulose due to affinity of their carbohydrate binding module (CBM) reduces their bulk concentration, and hence less enzyme is deactivated at the air-liquid interface. Devoid of substrate, shaking exposes more enzyme to the surface where they deactivate, and even 5 mg of Tween 20 was unable to completely avoid such deactivation in absence of substrate.

**Figure 5:**
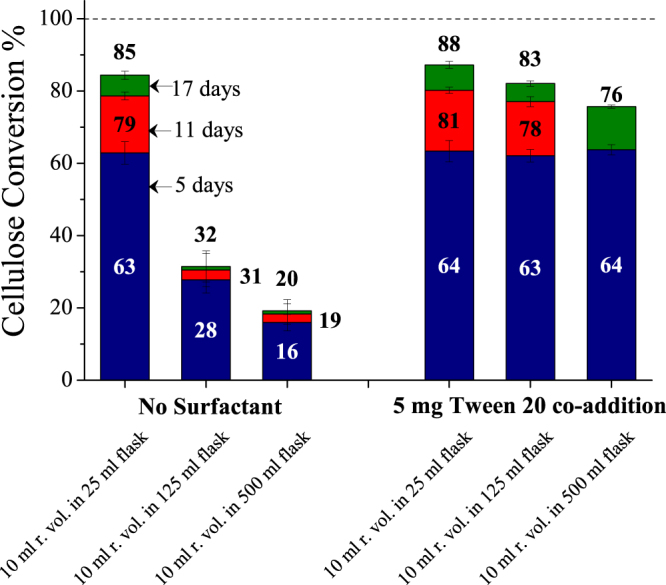
Effect of air-liquid interfacial area on enzymatic hydrolysis of Avicel with and without surfactant. Columns show cellulose conversion after 5, 11, and 17 days of enzymatic hydrolysis of Avicel (1% glucan loading) with 5 mg enzyme (Accellerase^®^ 1500) and with co-addition of 5 mg of Tween 20 added based on per gram glucan in substrate. Reaction volume was kept constant at 10 ml, and reactions were performed in 25, 125, and 500 ml Erlenmeyer flasks. Error bars represent standard deviation from three replicate flasks.

Because changing the interfacial area should affect cellulose conversions if the air-liquid interface causes deactivation, straightforward experiments were run with different interfacial surface areas by keeping the reaction volume constant but changing the size of reaction vessel. The approximate ratio of static interfacial area for 10 ml reaction volume in Erlenmeyer flasks of capacity 25:125:500 ml was 1:3.2:5.8. Figure 5 shows that high cellulose conversions were realized with low interfacial area and shaking (10 ml in 25 ml flask). Furthermore, when surfactant was added to a 10 ml reaction volume in a 500 ml reaction flask where the interfacial area was very high, cellulose conversions jumped up from 20 to 76%, clearly indicating that air-liquid interfacial area is the dominant cause of cellulase deactivation at low enzyme loadings. Also, considerable precipitation of solids was observed on walls of the 125 and 500 ml flasks that looked like a white ring at the highest point the liquid could reach in shaken flasks.

Why cellulase enzymes at low enzyme to substrate ratios cannot efficiently solubilize crystalline cellulose is a long-standing question. Based on published results including those by Whitaker *et al*.^15^, Reese^10^, Ooshima *et al*.^18^, Castanon and Wilke^11^, and Helle *et al*.,^13^ it wasn’t clear which mechanism is the prime driver for lowering of cellulase deactivation by surfactant. In light of current results, it is worthwhile to delve into the past work by Reese and co-workers on interfacial deactivation of cellulase. Reese and Mandels first introduced shaking induced cellulase inactivation in a May 1979 paper^43^ followed shortly by another paper^40^ by Reese and Ryu in October 1979 suggesting that shear may play the most important role in cellulase deactivation. In a March 1980 paper^10^, Reese proposed that shear and surface denaturation was minimized by a protein-surfactant complex that resists changes in protein confirmations. This paper was followed by a conference proceeding (June-July 1980) chapter^16^ that hypothesized three possibilities: 1) surfactants prevented shear-induced conformational change or aided in refolding of cellulase to native state; 2) shaking made it easier for proteases to change the cellulase confirmation; and 3) inactivation due to shaking is a surface related phenomenon. In their last article in June 1981^17^, Reese and co-workers showed far more severe cellulase deactivation in the presence of air bubbles than from shear stress alone in capillary tubing. However, the issue of cellulase deactivation and the role of surfactants took another turn when a few years later Sakata, Ooshima, and Harano (1985) challenged Reese’s results by showing no difference in rate of reaction or final conversion of Avicel cellulose by *Trichoderma viride* cellulase contained in 75 ml to 150 ml reaction volumes while stirring at 500 rpm at pH 4.8 and 40 °C in a cylindrical reactor. However, because the 5 wt% Avicel with 1 mg/ml cellulase used was equivalent to a high enzyme loading of 20 mg enzyme per g cellulose, their experiment did not show any difference in cellulose conversion with changing reaction volume. Next, Ooshima *et al*. in 1986 hypothesized that surfactants change the exoglucanase-endoglucanase ratio, while Castanon and Wilke^11^ in 1981 and Helle *et al*.^13^ in 1993 indicated that surfactants reduce enzyme immobilization to cellulose. Thus, further research was needed to build upon these findings for better understanding of cellulase deactivation. In this work, the use of experimental approaches far different from those applied by Reese in combination with conventional laboratory reactors (Erlenmeyer flasks) leave little doubt that deactivation of enzymes at the air-liquid interface are the primary cause of incomplete cellulose conversion at low enzyme loadings, consistent with the possible hypothesis first proposed by Reese and Mandels in 1979.

Proteins are known to deactivate at the air-liquid interface to minimize surface excess^44^. They can unfold and expose their hydrophobic groups to the gas phase that were previously buried inside their tertiary structure in bulk aqueous solution. Exclusion of hydrophobic regions from the aqueous phase decreases the free energy that drives their adsorption in the interfacial layer^44,45^. Because cellulases have a catalytic core and a carbohydrate binding module (CBM) joined together by a linker region^46^, it is possible for hydrophobic platform of CBM that binds to cellulose^47^ to orient itself toward the air phase and catalytic core toward the aqueous phase at the air-water interface. In this case, reorientation of the enzyme may make cellulase deactivation reversible. On the other hand, partial unfolding the cellulase that exposes the hydrophobic region in the active site of the catalytic core to the gas phase may be irreversible. Precipitation seen in large flasks at the low reaction volume suggests enzymes underwent aggregation in the interfacial layer. Such aggregations occur due to intermolecular hydrophobic interactions after their unfolding and is known to be irreversible for many proteins^48^. Dr. Reese also showed that cellulase deactivation was irreversible and found precipitation of protein in shaken flasks^16^. Therefore, it is more likely that the catalytic core undergoes unfolding rather than reorientation of CBM towards the gas phase.

Without shaking at an enzyme loading of 5 mg, the amount of enzyme exposed to the interfacial surface where it can be deactivated is too low to drop cellulose solubilization. However, shaking the flask at the same enzyme loading allows displacement of deactivated enzyme in this layer by active enzyme arriving from bulk solution, eventually making the ratio of deactivated to active enzyme large enough to affect cellulose conversion and reaction rate. Surfactants, due to their higher surface activity than cellulases can form a network of surface domains at the interface^49^, thereby reducing the amount of surface available for enzymes and the accompanying deactivation. This network was apparently stable in shaken flasks at conditions tested in this study. At high enzyme loadings, the amount of active enzyme is so large that most of the cellulose is hydrolyzed before enough enzyme can be deactivated to affect conversion. On the other hand, it is possible that unfolded enzymes initially adsorbed in the interfacial layer form a barrier^50^ and that prevents further enzyme deactivation while leaving enough enzymes in solutions at high loadings to still realize high conversions.

To further understand the responsible mechanism, enzymatic hydrolysis of lignocellulosic biomass was studied at low enzyme loading relevant to achieve low-cost sugar production for conversion to biofuels and renewable chemicals. As stated earlier, lignin can adsorb enzyme, and surfactants or proteins such as BSA block the lignin surface from adsorbing enzyme. Therefore, dilute acid (DA) and CELF pretreatments were applied to BESC standard poplar solids to leave high and low amounts of lignin in the pretreated solids, respectively. In particular, dilute acid pretreated poplar solids contained 64% glucan, 2% xylan, and 28% Klason lignin (acid insoluble lignin) while solids from CELF pretreatment of poplar contained 90% glucan, 3% xylan and 4% Klason lignin. A significant increase in enzymatic hydrolysis of the cellulose in DA pretreated poplar was only evident when a large amount of surfactant (100 mg Tween 20) was added, consistent with surfactant blocking the lignin surface from adsorbing enzyme. However, in the CELF pretreated poplar solids containing much less lignin, addition of just a small amount of surfactant (5 mg Tween 20) increased the yield from 55 to 85%. It is important to note that hydrolysis yields after 11 days of reaction without shaking and without surfactant were similar to those from shaking combined with a low amount of surfactant for low-lignin lignocellulosic biomass but with the drawback of slower reaction rate, similar to the results seen with Avicel cellulose. Enzyme added to a mixture of lignocellulosic biomass can bind to cellulose, lignin, or hemicellulose but to a lesser extent, remain in bulk solution, reside at the interface to lower the free energy of the gas-liquid interface, and bind in limited quantities to the solid-liquid interface. Furthermore, the concentration of enzyme on the cellulose surface, in the bulk solution, and at the interface change with time reaction. When low amounts of a surface-active additive are added to biomass solids with high hydrophobic lignin content such as that from dilute acid pretreatment of poplar for hydrolysis at low enzyme concentrations, most of the surfactant is bound to lignin, with little left at the interface. As a result, enzymes still deactivate at the gas-liquid interfacial phase boundary, and the slight reduction in unproductive binding of enzyme to lignin has a miniscule effect on cellulose conversion due to the fractional blocking of lignin by surfactant. On the other hand, biomass solids with low lignin content produced by CELF pretreatment offer a large number of cellulose binding sites while limiting the amount of lignin available for unproductive enzyme binding. Thus, at low enzyme concentrations, addition of surfactant or absence of shaking reduces interfacial deactivation of enzymes and increases active enzyme concentrations that allow large increase in cellulose conversion, similar to that seen for Avicel cellulose. Moreover, this mechanism explains why BSA supplementation generally increased sugar yields from enzymatic hydrolysis of flowthrough pretreated poplar solids with lower lignin content than batch pretreated poplar solids with higher lignin content^4^, contrary to the expectation that blocking of lignin by BSA would have had a larger effect on cellulose conversion of batch pretreated poplar.

The high foaming of globular protein BSA^51^ results in strikingly similar effects to the surfactant Tween 20. BSA lowers the surface tension of water from 72.5 to about 50 ergs/cm^2 ^^52^. Like BSA, human serum albumin (HSA) is also highly surface active, and accumulates at the air-liquid interface in a denatured but reversible state. These albumins and surfactants orient their hydrophobic moieties towards the air (gas) phase to reduce solution surface tension^53^. Additives such as polyethylene glycols and polypropylene glycols lower surface tension of water, and their surface activity increases with concentration and degree of polymerization^54^. The earlier use of term “amphiphilic additive” in this work could alternatively be labeled as “surface-active additives” or “surface-active agents” as they reduce cellulase deactivation at the air-liquid interface.

In the natural environment of wood-rot fungi, small concentrations of extracellular cellulases are capable of slowly degrading cellulose in wood over the course of several weeks without shaking as applied in laboratory practice^55^. On the other hand, commercial enzyme preparations consist of *T. reesei* secretome supplemented with *β*-glucosidase as the fungus does not naturally produce enough *β*-glucosidase to convert cellobiose to glucose fast enough to prevent cellulose inhibition^7,56,57^. None of our extensive experiments showed increased cellobiose concentrations that would indicate *β*-glucosidase deactivation at the interface. However, proteomic analysis of the commercial cellulase preparations used for deconstruction of lignocellulosic biomass show a broad array of enzyme activities: roughly 40% cellobiohydrolases, 20% endoglucanases, 15% *β*-glucosidases, 5% endoxylanases, 6 to 10% xyloglucanases, less than 2% each of acetyl xylan esterases, *α*-arabinofuranosidases, *α*-glucuronosidase, and 10 to 15% of non-cellulolytic enzymes and low-abundance glycosyl hydrolases^58^. Two studies indicated that cellobiohydrolase II (CBHII; also known as exoglucanase II or Cel6A), maybe susceptible to deactivation. A 1997 article^59^ suggested that CBH II immobilization on cellulose is reduced by Tween 20, but their preparation had 90% CBH II from *T. viride*. Another recent article^60^ indicated that CBH II is only stabilized by Tween 80 at 30 °C under agitation conditions, but despite finding agitation to be a decisive factor, the authors did not offer a mechanism that could explain this effect. These two studies provide preliminary evidence that CBH II might be the component in enzyme cocktail that is affected, but, in any case, the reason for its deactivation is unfolding at the interface to minimize surface tension for achieving thermodynamic stability. However, commercial enzyme preparations contain higher amounts of CBH I than CBH II, and a relatively high amount of endoglucanase I (EG I)^58^. Since cellulose conversions were at least 40 percentage points lower after 17 days when enzymes could liberally move to the interfacial layer and deactivate in the absence of surfactant, this level of loss in cellulose conversion indicates CBH I and EG I, and not just CBH II, may also be susceptible to deactivation. At this point, it is uncertain which component is deactivated most at the interface as it is difficult to obtain pure enzymes in the amounts needed for such a study. A further improvement can be made in targeting mass loadings of surfactant by determining its critical micelle concentration (CMC) in the hydrolysis environment. CMC can be affected by substrate and enzyme concentration, pH, ionic strength due to buffer and antibiotic, temperature, and time-dependent glucose concentration in the liquid from reaction of solid substrate. A concentration that realizes a minimum in surface tension is required, beyond which, the it will only accumulate as micelles in the bulk solution and increase the cost of hydrolysis of lignin-free cellulosic substrate at low enzyme loading.

Cellulases appear to fall in the category of air-sensitive proteins like lysozyme and BSA and peptides such as insulin^61^ and recombinant human growth hormone (rhGH)^62,63^ and need to be further explored. Since enzymes stock solutions are almost always shaken to evenly distribute them before taking aliquots for experiments, adding a small quantity of non-ionic or biological surface-active agent to current commercial enzyme preparations might improve enzyme shelf-life. These experiments show that cellulases deactivate at the air-liquid interface to lower free energy, resulting in incomplete Avicel hydrolysis at low enzyme loading. Thus, the results in this paper showed that of ten possible mechanisms, the one first proposed by Reese and co-workers in 1980s is primarily responsible for cellulase deactivation at low enzyme loadings. Moreover, cellulase deactivation at the air-liquid interface significantly reduced hydrolysis of cellulose in solids with low lignin content at low enzyme loadings in addition to pure cellulose. As a result, simply reducing the air-liquid interface of the cellulose-cellulase system through selection of the proper size glassware for shaking can dramatically improve cellulose conversion at low enzyme loadings. And adding small amounts of surface-active agents that do not denature the enzyme and are compatible with downstream fermentation microbes could significantly lower activity loss by reducing cellulase accumulation at the air-liquid interface.

## Methods

### Materials

Avicel (Avicel^®^ PH-101, Fluka, Cat. No. 11365-1KG, Lot No. BCBN7864V), bovine serum albumin (BSA) (Cat. No. A7906-500G, Batch No. 078K0730), and defatted soybean flour (Cat. No. S9633, Lot No. SLBL7333V, 52% approximate protein content and 85+% dispersible and 1% fat as noted by the manufacturer) were purchased from Sigma-Aldrich Corp. at St. Louis, MO. Tween^®^ 20 was purchased from Acros Organics (Lot No. A0226412). Corning^®^ Pyrex^®^ 125 ml Erlenmeyer flasks made of borosilicate glass (Cat. No. 4985–125), 25 ml Erlenmeyer flasks made of borosilicate glass, Kimble^®^ Kimax^®^ Valueware^®^, (Cat. No. 5650025EMD), 125 ml Erlenmeyer flasks made of polycarbonate material Fisherbrand (Cat. No. PBV125), Corning^®^ Pyrex^®^ 500 ml Erlenmeyer flasks made of borosilicate glass (Cat. No. 4995-500), tetrahydrofuran (certified) and 72 w/w% sulfuric acid (Ricca Chemical) were all purchased through Fisher Scientific, Thermo Fisher Scientific Inc. at Waltham, MA. Accellerase^*®*^ 1500 (Batch No. 1662334068), was a kind gift from DuPont Industrial Biosciences at Palo Alto, CA. Due to their high foaming tendency, 1% stock solutions of BSA or Tween 20 were made by weighing 1 gram of BSA or Tween 20 in a tared 125 ml conical flask followed by addition of milli-Q water to reach a total mass of 100 grams for accuracy and reproducibility. For 1% soy protein stock solution, 2.3 grams of soy flour (considering protein content of 52% and 85% dispersion) was weighed in a similar conical flask and brought to a total mass of 100 grams by adding milli-Q water. For experiments with glass powder (enhanced shear/high solid surface area), a 20 ml borosilicate glass serum vial (Wheaton^®^ Cat. No. 223687) was crushed with a metal hammer until the particle size was about 0.250 mm as determined by passing through a no. 60 sieve (W.S. Tyler ASTM E11-09). Although some of the powder was very fine, the larger and small particles were easily separated by gravity. 0.25 g each of the large and fine sizes were added to Erlenmeyer flask after addition of Avicel.

BESC (Bioenergy Science Center) standard poplar was provided by the National Renewable Energy Laboratory (NREL), Golden, CO as chips and knife milled through a 1 mm screen (Model 4 Wiley Mill Thomas Scientific, Swedesboro, NJ) at the University of California Riverside. Batch dilute acid (DA) and co-solvent enhanced lignin fractionation (CELF) pretreatments were applied to 10% poplar solids loading in a 1 L Parr® reactor (Parr Instruments, Moline, IL, USA) with a total reaction mass of 800 g. Dilute acid pretreatment of biomass was carried out with 0.5 wt% sulfuric acid in water at 160 °C for 25 minutes. CELF pretreatment of the same biomass was performed at 160 °C for 15 minutes in a mixture of equal volumes of 0.5 wt% sulfuric acid in water and tetrahydrofuran solvent. Pretreated solids were vacuum filtered with Whatman^®^ glass microfiber filter and thoroughly washed with DI water. An elaborate description of the pretreatment procedure has been previously documented^64^.

### Reaction vessel preparation

All flasks were first thoroughly scrubbed and cleaned with lab detergent, followed by several washings with tap water and finally with deionized water, and dried at 105 °C for 24 hours. For siliconized glass experiments, 125 ml Erlenmeyer flasks were siliconized with Aquasil^®^ siliconizing fluid (Cat. No. TS42799, Thermo Scientific, Thermo Fisher Scientific Inc.). Freshly prepared 1% Aquasil solution in Milli-Q water was added to flasks up to 125 ml mark and shaken for 15 seconds. The solution was drained and the flasks were washed twice with 100% methanol (Fisher Scientific, Thermo Fisher Scientific Inc.) and dried at 105 °C for 6 hours.

### Compositional analyses

Composition of Avicel cellulose as determined by the standard NREL procedure “Determination of Structural Carbohydrates and Lignin in Biomass” was 97% glucan and 3% xylan by mass^21^. Avicel had an average 4 wt. % moisture content as determined by a halogen moisture analyzer (HB43-S; Mettler-Toledo, Columbus, OH). Wet pretreated solids were dried at 40 ± 1 °C in an incubator for several days until the moisture content dropped to about 5% before compositional analysis.

### Enzymatic hydrolyses

**Figure 6:**
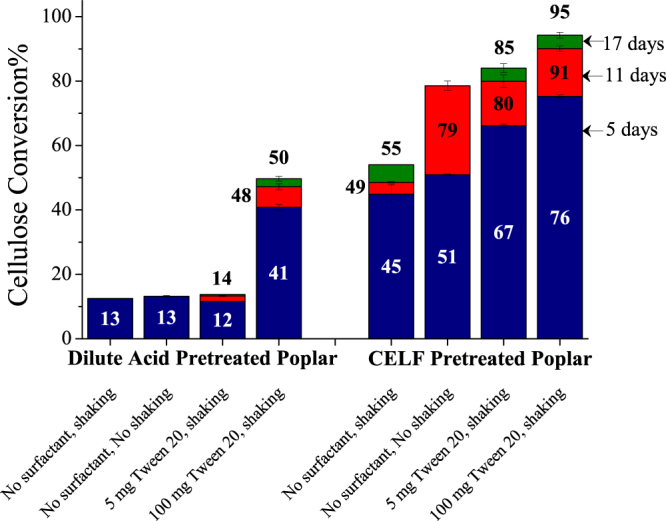
Effect of surfactant and shaking on enzymatic hydrolysis of dilute acid and CELF pretreated poplar. Columns show cellulose conversion after 5, 11, and 17 days of enzymatic hydrolysis of dilute acid (0.5% H_2_SO_4_) and CELF (0.5% H_2_SO_4_ in 1:1 H_2_O:THF) pretreated poplar (1% glucan loading) with 5 mg enzyme (Accellerase^®^ 1500) with and without shaking at 150 rpm, and with co-addition of 5 mg or 100 mg of Tween 20 added based on per gram glucan in substrate. Error bars represent standard deviation from three replicate flasks.

All enzymatic hydrolysis runs were according to the NREL standard procedure “Enzymatic Saccharification of Lignocellulosic Biomass”^65^ with only the following modifications. All experiments were done at 1 w/v% glucan loading of Avicel or pretreated poplar. Never-dried pretreated poplar solids were used for enzymatic hydrolysis to avoid drying possibly collapsing pores and thereby lowering cellulose accessibility. The reaction volume was 50 ml performed in 125 ml Erlenmeyer flasks in all experiments except those evaluating the effect of interfacial area (Fig. 5) for which 10 ml of reaction volume was carried out in 25, 125, or 500 ml Erlenmeyer flasks. The static interfacial areas measured with a ruler or Vernier calipers of 10 ml reaction volume for 25, 125, and 500 ml flasks were 7.9 cm^2^, 25.6 cm^2^, and 45.6 cm^2^, respectively. All experiments were carried out in reaction vessels made of borosilicate glass except those evaluating the effect of reaction vessel surface or shear (Fig. 3) for which siliconized borosilicate glass flasks, polycarbonate flasks, and borosilicate glass flasks containing 0.5 grams of glass powder were used along with borosilicate glass flasks. All enzymatic hydrolyses were carried out in 50 mM sodium citrate buffer at pH 5.0, 50 °C, and 150 rpm for shaking experiments and 0 rpm for those without shaking. Orbital shaking was in a Multitron Standard (Infors^®^ HT Biotech, Laurel, MD) that held the flasks firmly with sticky pads. In addition to the temperature reading visible in the LCD panel, a K-type thermocouple (Omega Engineering Co., Stamford, CT) was inserted inside the shaker in several locations as a secondary measure to assure temperature stability. Accellerase^®^ 1500 with a BCA protein content of 82 mg/ml^23^ was diluted 20 times to 4.1 mg/ml in 50 mM citrate buffer in 100 ml borosilicate glass volumetric flasks. Accellerase^®^ 1500, BSA, Tween 20, and soy loadings were based on milligrams of protein or surfactant per gram glucan in the substrate. In all experiments including no shaking experiments, the reaction medium was shaken gently after addition of enzyme. In the co-addition experiments, BSA, Tween, or soy was added to the Erlenmeyer flask quickly after the enzyme of (co-addition). Loading of Accellerase^®^ 1500 was either 5 or 30 mg protein per gram glucan in Avicel. 5 mg or 100 mg BSA or Tween 20 was also added based on grams of glucan in Avicel. Loading of Accellerase^®^ 1500 was 5 mg protein per gram glucan in unpretreated poplar for lignocellulosic biomass experiments (Fig. 6). Glucan yields from the pretreatment process were 96% and 100% for DA and CELF pretreatments, respectively, making enzyme loading in enzymatic hydrolysis flask as 5.2 mg for DA and 5 mg for CELF on per gram glucan basis. This enzyme loading method involving a pretreatment process have been documented previously^64^. 5 mg or 100 mg Tween 20 was added based on grams of glucan in pretreated poplar. In cellulose conversion vs. time experiments (Fig. 1), sampling for 5 mg cellulase, co-addition of 5 mg BSA and 5 mg cellulase, 30 mg cellulase, co-addition of 30 mg cellulase and 100 mg BSA, were at 4, 24, 48, 72, 96, 120, 168, 216, 264, and 408 hours after enzyme addition. For all other conditions, sampling was at 120, 264, and 408 hours.

Enzymatic hydrolysis results for all conditions were determined from three replicates in Erlenmeyer flasks. Error bars in all figures represent sample standard deviation from three replicate flasks. For no shaking experiments, separate flasks were kept for each time point (120, 264, and 408 hours) to not to disturb the reaction medium. Sampling was by withdrawing a 0.5 ml homogenous aliquot followed by centrifugation in a fixed-angle centrifuge (Eppendorf^®^ Microcentrifuge Model 5424, Eppendorf North America, Hauppauge, NY) at 15000 rpm for 5 minutes. The supernatants were analyzed on a Waters^®^ e2695 Separations Module with detection on Waters^®^ 2414 RI detector (Waters Corp., Milford MA) equipped with a Bio-Rad^®^ Aminex^®^ HPX-87H column conditioned at 65 °C using 5 mM sulfuric acid mobile phase at a flow rate of 0.6 ml/min for all separations. Cellulose conversion was calculated by:

$$\begin{array}{c}{\rm{Cellulose}}\,{\rm{conversion}}\,({\rm{glucan}}\,{\rm{yield}} \% )\,=\\ \,\frac{({\rm{Glucose}}\,(\frac{{\rm{mg}}}{{\rm{ml}}})+({\rm{Cellobiose}}(\frac{{\rm{mg}}}{{\rm{ml}}})\ast 1.053))\,\ast \,{\rm{Reaction}}\,{\rm{volume}}\,({\rm{ml}})\,\ast \,0.9\,\ast \,100}{{\rm{Glucan}}\,{\rm{in}}\,{\rm{cellulosic}}\,{\rm{substrate}}\,({\rm{mg}})}\end{array}$$

where 0.9 accounts for the mass of water added to cellulose (glucan) during enzymatic hydrolysis and 1.053 accounts for the addition of water to form glucose from cellobiose. A soy flour blank was also kept along with other flasks that contained 5 mg soy flour and 5 mg Accellerase 1500 similar to other experiments with substrate and soy as additive. An HPLC chromatogram showed no carbohydrates or any other soy flour derived components to be present. In the experiments that studied effect of interfacial area (Fig. 5), aliquots from 10 ml reaction volume in 500 ml flask in presence of surfactant after 11 days of reaction could not be analyzed. Thus, only cellulose conversion after 5 and 17 days of reaction are shown for this condition. Sampling could not be done for no surfactant and no shaking condition of CELF pretreated poplar (Fig. 6) after 17 days of reaction, and only conversions after 5 and 11 days of reaction are shown.

### Data Availability

All data generated or analyzed during this study are included in this published article.
